# Editorial: The synthesis of secretory immunoglobulin A in mucosal tissue: mucosal-associated invariant T, T follicular helper, and B cells

**DOI:** 10.3389/fimmu.2024.1504432

**Published:** 2024-10-17

**Authors:** Jayaum S. Booth, Rezwanul Wahid, Dunja Bruder, Rosangela Salerno-Goncalves

**Affiliations:** ^1^ Center for Vaccine Development, University of Maryland, Baltimore, MD, United States; ^2^ Institute of Medical Microbiology and Hospital Hygiene, Otto-von-Guericke University Magdeburg, Magdeburg, Germany; ^3^ Immune Regulation Group, Helmholtz Center for Infection Research, Braunschweig, Germany

**Keywords:** MAIT cells, T follicular helper (Tfh) cell, B cells, IgA, mucosal immune system (MIS)

The mucosal immune system (MIS) plays a fundamental role in maintaining and protecting the intestine against pathogens. Immunoglobulin A (IgA) is a key component of the MIS and the predominant immunoglobulin isotype found in mucosal secretions (1, 2). Beyond its role in neutralizing pathogens, IgA is essential in maintaining intestinal homeostasis, shaping the microbiome, and influencing systemic immunity (3, 4). Mucosal-associated invariant T (MAIT) cells, a type of innate-like T cell, populate mucosal sites, and help modulate the MIS, offering protection against microbial threats (5). The differentiation of T cell-dependent IgA-secreting B cells in gut-associated lymphoid tissues requires T follicular helper (T_FH_) cells, which provide critical signals through cytokines and surface molecules (6, 7).

Understanding the interplay of MAIT, B, and T_FH_ in producing IgA has significant implications for various health conditions, including autoimmune diseases, allergies, and infections. Recent studies have demonstrated functional interactions between these cell types, suggesting well-coordinated crosstalk within mucosal immune compartments, leading to enhanced secretory IgA (SIgA) production and/or improved MAIT cell function. For example, Leung’s group demonstrated both *ex vivo* and *in vivo* the role of MAIT cells in promoting B cell differentiation into plasmablasts and subsequent IgA production (8). Additionally, Salerno-Gonçalves and colleagues found that B cell upregulation of HLA-G expression downregulates the inhibitory HLA-G receptor, CD85j, on MAIT cells, leading to MAIT cell loss (9). More recently, Pankhurst et al. discovered that MAIT cells activate dendritic cells to promote T_FH_ activity, enhancing antigen-specific SIgA production in a mouse model of influenza A infection (10). Despite these insights, key knowledge gaps remain regarding the interactions and contributions of MAIT, T_FH_, and B cells to SIgA production at mucosal sites. Our research topic, comprising one original research article and three review manuscripts, contributes to this understanding by exploring how these interactions function in IgA production and how they may be altered during homeostasis disruption

In their original research article, Hattori-Muroi et al. demonstrated that oral administration of voglibose, an α-glucosidase inhibitor (α-GI) commonly used to reduce post-prandial blood glucose levels in diabetic patients, enhances IgA production in Peyer’s patches in mice. This effect was mediated by increasing interactions between T_FH_ and germinal center B cells and eventually was linked to changes in the gut microbiota rather than glucose reduction. These novel findings suggest that voglibose may promote the colonization of *Bifidobacteriaceae* and/or inhibit the colonization of *Lactobacillaceae*, thereby altering the microbiome composition and function. Additionally, the authors demonstrated that voglibose significantly increased SIgA production against attenuated *Salmonella* Typhimurium, highlighting its potential as an adjuvant at mucosal sites. This elegant research article is a valuable contribution to the ongoing pursuit of developing next-generation mucosal adjuvants, which are crucial for advancing needle-free vaccine delivery methods—such as nasal or oral administration—to enhance patient compliance in mass vaccination programs.

The review by Seefeld et al. explores innovative engineering strategies aimed at enhancing mucosal uptake through active targeting and passive transport, immune activation, and regulation within the respiratory mucosa. The authors offered a comprehensive overview of the anatomy of the upper and lower respiratory tracts, along with their associated lymphoid tissues, emphasizing the critical role of IgA in mucosal immunity within these regions. Additionally, they explored preclinical mucosal vaccine approaches currently under development and discussed potential strategies for targeting the respiratory tract to induce immune tolerance. The review highlights how a deeper understanding of nasal-associated lymphoid tissue (NALT) and bronchial-associated lymphoid tissue (BALT) biology can inform the development of more effective strategies for immune activation and tolerance modulation. This review is particularly timely, not only in light of the recent pandemics caused by the 2009 influenza A (H1N1) (11) and 2019 SARS-CoV-2 (12) viruses, but also due to the ongoing global battle against respiratory diseases such as tuberculosis and influenza.

A timely review by Carreto-Binaghi et al. offers a fresh perspective on IgA’s role in the intestinal mucosa. The authors emphasize IgA’s critical function in maintaining homeostasis and protecting against enteric bacterial pathogens. They not only highlight the interplay between MAIT, T_FH_, and B cells in IgA production, but also explore the contributions of other cell subsets, such as regulatory T cells (Tregs), innate lymphoid cells (ILCs), dendritic cells (DCs), macrophages (MΦ), eosinophils, and epithelial cells. These cells contribute directly or indirectly to IgA production, forming a complex, coordinated immune network essential for maintaining mucosal homeostasis. The review also discusses the role of long-lived memory B cells and antibody-secreting cells in sustaining IgA production, as well as how vaccination and infection influence the long-term maintenance of pre-existing IgA. Understanding the impact of these long-lived cells is essential for developing effective oral vaccines. Additionally, insights into how prior infections or vaccinations affect the long-term presence of IgA can help guide the design of vaccines that leverage pre-existing immunity to strengthen protective responses at mucosal surfaces.

MAIT cells are conventionally known for their anti-infective activities against bacteria and viruses. This review by Gao et al. explores recent discoveries regarding additional functions of MAIT cells, including their roles in maintaining and repairing local tissues, such as the intestinal mucosa, meningeal barrier, and skin. The authors provided a systematic review of their phenotypes and targeted their chemotaxis in context to their local tissue residence, maintenance, and immunoregulatory role in wound healing. Furthermore, the authors included a perspective on the future implementation of the homeostatic functions of MAIT cells in developing therapies for chronic inflammation and/or restoring tissue integrity. There are potential challenges to overcome, particularly in obtaining human mucosal tissues and developing *ex vivo* research models that accurately simulate *in vivo* disease environments, among others. It is worth highlighting that intranasal co-administration of protein antigens with a strong MAIT cell ligand stimulates mucosal IgA production, positioning MAIT cells as a potential cellular adjuvant for enhancing humoral immunity for vaccine development (13).

We believe that this Research Topic offers an updated perspective on the contribution of MAIT, T_FH_, and B cells to the production of IgA and the development of advanced vaccines and targeted intervention strategies.
